# CircAGFG1 promotes cervical cancer progression via miR-370-3p/RAF1 signaling

**DOI:** 10.1186/s12885-019-6269-x

**Published:** 2019-11-08

**Authors:** Fengqin Wu, Jingjing Zhou

**Affiliations:** 1 Department of Gynecology, Shangluo Central Hospital, Shangluo City, 726000 Shaanxi Province China; 2 Department of Gynaecology, Ankang Hospital of Traditional Chinese Medicine, No.47, Bashan Road(east), Hanbin District, Ankang City, 725000 Shaanxi Province China

**Keywords:** circAGFG1, miR-370-3p, RAF1, Cervical cancer

## Abstract

**Background:**

In past decades, circular RNAs (circRNAs) have achieved increasing attention because of its regulatory role in different kinds of cancers. However, how circAGFG1 regulates cervical cancer (CC) is still largely undiscovered. This study aims to evaluate the role of a novel circRNAs and related molecular mechanism in CC cells.

**Methods:**

High or low level of circAGFG1 was detected in CC cells or normal cell line with qRT-PCR. The proliferative and migratory abilities of CC cells were assessed with loss-of function assays. The downstream miRNA and mRNA of circAGFG1 were searched out and proved by using bioinformatics analysis and mechanism experiments. Recue assays were designed to confirm the role of circAGFG1/miR-370-3p/RAF1 axis in CC cell activities.

**Results:**

The levels of circAGFG1 was abundant in CC cells in comparison with normal cervical cell End1/E6E7. The inhibitory effect of decreased circAGFG1 level on the proliferative and migratory abilities of CC cells was assessed. CircAGFG1 and miR-370-3p were localized in the cytoplasm and they can interact with each other. Moreover, miR-370-3p was downregulated in CC cells. We also determined the negative effect of miR-370-3p on RAF1. CircAGFG1 could promote RAF1 expression by absorbing miR-370-3p, thereby activating RAF/MEK/ERK pathway. circAGFG1 promoted proliferation and migration of CC cells via enhancing the activity of RAF/MEK/ERK pathway by sponging miR-370-3p and further regulating RAF1.

**Conclusion:**

The results of this study provided new evidence that circAGFG1 acted as a vital regulator in cervical cancer proliferation and migration, giving great promise to apply it as a potential biomarker for diagnosis and therapy in CC treatment.

## Background

According to global cancer statistics in 2018, cervical cancer (CC) is acknowledged as the 2nd most commonly-diagnosed tumor, whose fatality rate was the 2nd for female [1]. More than 570,000 patients were diagnosed with CC, and 311,000 death cases were reported in the past year. Considerable advances in treatment have been made over the past decades. However, mortality rate of CC remains high for lagging diagnosis, which was owing to the lack of clear cancer biomarkers [2]. Therefore, discovering molecular mechanisms in CC progression and finding effective therapeutic targets are urgently needed.

Circular RNAs (circRNAs), a newly-discovered non-protein coding RNAs, are featured with a continuous closed loop with no 3′-poly A tail as well as 5′-cap structure [3]. CircRNAs are mostly discovered to mediate gene expression in cancer development through sponging competitive regulators, especially microRNAs (miRNAs) [4]. Recently, mounting evidence has proved that circRNAs can regulate cervical cancer progression via the ceRNA network. For example, circRNA hsa_circRNA_101996 induced the upregulation of TPX2 by restraining miR-8075 to promote CC proliferation and migration [5]. Knockdown of circular RNA hsa_circ_0000263 regulates miR-150-5p/MDM4/p53 pathway and inhibits CC progression [6]. Circ_0067934 modulates miR-545/EIF3C axis to stimulate CC progression [7]. CircRNA8924 serves as an oncogene to facilitate proliferation and migration of CC cells by regulating CBX8 expression via sequestering miR-518d-5p/519-5p family [8]. In our research, CircRNA circAGFG1 with ID of hsa_circ_0058514 (chr2:228356262–228,389,631; circBase: http://www.circbase.org/cgi-bin/simplesearch.cgi) was selected for investigation. CircAGFG1 has been proved to be a facilitator in the progress of triple-negative breast cancer by absorbing miR-195-5p and modulating CCNE1 expression [9]. And circAGFG1 exhausts miR-203 to increase the expression of ZNF281 thereby boosting metastasis of non-small-cell lung cancer [10]. However, its effect on cervical cancer and associated mechanisms in CC have not been totally discovered.

In our research, circAGFG1, miR-370-3p and RAF1 constituted a ceRNA network to regulate cervical cancer cellular processes. MiR-370-3p has been indicated to participate in pancreatic cancer, glioblastoma and bladder cancer and so on [11–13]. Serine/threonine kinase (RAF1), also names Raf-1 proto-oncogene, is a well-known oncogene in multiple carcinomas, such as lung cancer, glioma and gastric cancer [14–16]. The correlation among these three genes in cervical cancer is unclear.

In the present study, we first found that circAGFG1 was upregulated in CC and circAGFG1 silencing inhibited the proliferation and migration abilities. We also discovered that circAGFG1 promoted RAF1 expression by sponging miR-370-3p and further activated RAF/MEK/ERK pathway to regulate CC progression. Our study showed that circAGFG1 promoted cervical cancer progression via miR-370-3p/RAF1/MEK/ERK signaling. These data may offer a potent diagnostic biomarker and a novel biological target for CC treatment.

## Methods

### Cell culture

American Type Culture Collection (ATCC; Manassas, VA, USA) was the institute provided cell lines used in these study on 4th, Nov, 2018. Cell lines used in these study including: HeLa (Cat: CCL-2), C-33a (Cat: HTB-31; Cat: CRL-2615), SiHa (Cat: HTB-35), HCC94 and End1/E6E7 (normal cervical cell line). Cells were grown in Dulbecco’s Modified Eagle’s Medium (DMEM; Gibco, Carlsbad, CA, USA) added with 10% fetal bovine serum (FBS; Gibco), 100 U/mL penicillin and 100 mg/mL streptomycin, which were then maintained in a humidified air at 37 °C with 5% CO_2_. The culture medium was refreshed every 2 days. Cell lines used in this study had been authenticated by STR cell identification on 13th, Nov, 2018. Cell lines were not infected by mycoplasma. All experiments were performed with mycoplasma-free cells. Cells were not contaminated when referring to NCBI.

### Cell transfection

All plasmids including siRNAs against circAGFG1 containing si/circAGFG1#1 (reduced circAGFG1 expression by 87% in HeLa cells and by 84% in SiHa cells), si/circAGFG1#2 (reduced circAGFG1 expression by 81% in HeLa cells and by 79% in SiHa cells), si/circAGFG1#3 (reduced circAGFG1 expression by 75% in HeLa cells and by 71% in SiHa cells), pcDNA3.1/circAGFG1 (increased circAGFG1 expression to about 114 fold change in HeLa cells and to about 108 fold change in SiHa cells), pcDNA3.1/RAF1 (increased RAF1 expression to about 123 fold change in HeLa cells), miR-370-3p mimic (increased miR-370-3p expression to about 104 fold change in HeLa cells and to about 111 fold change in SiHa cells) and miR-370-3p inhibitor (reduced miR-370-3p expression by 79% in HeLa cells and by 72% in SiHa cells) were constructed. All plasmids and their negative controls were purchased from GenePharma (Shanghai, China) and transfected into CC cells with Lipofectamine® 2000 (Invitrogen) under recommended direction.

### qRT-PCR

Total RNA extraction was conducted with TRIzol reagent (Invitrogen), which were reverse-transcribed into cDNA by utilizing PrimeScript RT Reagent Kit (Takara, Dalian, China) routinely. qRT-PCR were carried out by using TB Green Premix Ex Taq (Takara) on the Bio-Rad CFX96 system (Bio-Rad, CA, USA). Quantification of circRNA and mRNA and miRNA was made by referring to GAPDH or U6. Relative gene expression was determined with the use of 2^–ΔΔCT^ method. Special primers are listed as follows:

circAGFG1:

F: 5′-CCAGTTGTAGGTCGTTCTCAAG-3′.

R: 5′-GGATTTAATCCTCGCCTGCATG-3′.

miR-370-3p:

F: 5′-TGTAACCAGAGAGCGGGATGT-3′.

R: 5′-TTTTGGCATAACTAAGGCCGAA-3′.

RAF1:

F: 5′- CTTCAGGAACGAGGTGGCTGTT-3′.

R: 5′- TGCTGCCTTCACACCACTGAGT-3′.

GAPDH:

F: 5′-GAAGGTGAAGGTCGGAGTC-3′.

R: 5′-GAAGATGGTGATGGGATTTC-3′.

U6:

F: 5′-CTCGCTTCGGCAGCACA-3′.

R: 5′-AACGCTTCACGAATTTGCGT-3′.

### Cell viability and proliferation assays

1 × 10^3^ CC cells were maintained in 96-pore plates for 1 d and grown for extra 0, 24, 48, 72 and 96 h. Cell Counting Kit-8 (Bosterbio, Wuhan, China) was utilized to evaluate cell viability following the supplier’s protocol at each time point. After 4 h of culture, a spectrophotometric plate reader (BioTek, VT, USA) was applied to measure the optical density at 450 nm for detection of cell viability.

CC cells (200 μL, 2 × 10^4^/mL) were immobilized by 70% alcohol and subsequently cultured with 50 μM EdU (5-Ethynyl-2′-Deoxyuridine) labeling solution (Invitrogen) for 2 h at 37 °C. The fluorescent intensity of EdU was examined at 550 nm through applying Cell Light EdU DNA imaging kit (Invitrogen). Cells were cultivated using 5 μg/mL Hoechst 33342 for 0.5 h for DNA staining. The visualization and photograph of immunostainings were implemented with a fluorescent microscope (Olympus inverted microscope IX71).

### Flow cytometry analysis

Apoptosis of indicated cells was measured using flow cytometry analysis in accordance with a previous study [17].

### Transwell migration assay

Cells with a density of 1 × 10^5^ cells/hore were seeded in 6-well plates, suspended in 200 μL serum-free medium and planted into the upper chambers without Matrigel mixture. 500 μL medium with 10% FBS was added into the lower chambers (BD BioCoat, MA, USA) as attractants. After 24 h, cells in the upper chambers were removed. Migrated cells in the lower chamber were fixed by ethanol and stained with crystal violet, which were subsequently photographed and calculated by a light microscope (Olympus Corporation, Tokyo, Japan).

### Fluorescence in situ hybridization (FISH)

Specific Cy3-labeled circAGFG1 probe and FITC-labeled miR-370-3p probe were designed and synthesized via RiboBio (Gangzhou, China) at 37 °C for whole night, and dyed utilizing DAPI obeying the guidebooks of the supplier. Slides were photographed with a fluorescence microscope (Leica, Wetzlar, Germany).

### Luciferase reporter assay

Binding sequences of circAGFG1 and RAF1 3′UTR for miR-370-3p as well as mutant versions (circAGFG1-WT, RAF1-WT; circAGFG1-MUT, RAF1-MUT) were synthesized and subcloned into luciferase reporter vector pGL3 (Promega, Madison, WI, USA). Then these vectors were co-transfected with NC/mimic or miR-370-3p mimic into HeLa and SiHa cells. For affirming the specificity of miR-370-3p and circAGFG1, we constructed the full length of circABCC2, circLRP6, circSCAF11 and circAGFG1 into luciferase reporter vector pGL3 to observe the changes of these luciferase activities by miR-370-3p mimics. As for the regulation of miR-370-3p and circAGFG1 on the luciferase activity of RAF1, RAF1-WT or RAF1-MUT was co-transfected with NC/mimic, miR-370-3p mimic, miR-370-3p mimic + pcDNA3.1 or miR-370-3p mimic + pcDNA3.1/circAGFG1 into CC cells. The luciferase activities were measured by Dual Luciferase Assay Kit (Promega) according to the manufacturer’s directions.

### RNA immunoprecipitation (RIP)

Under the manufacturer’s protocols, RIP was performed with Magna RIP kit (Millipore). Transfected cells were lysed in RNA lysis buffer and cell lysates were incubated with magnetic beads with anti-Argonaute2 (Ago2) or anti-IgG (both from Millipore) at 4 °C for 4 h. After the beads were washed, the immuno-precipitated RNAs were purified and detected by qRT-PCR.

### RNA pull-down assay

G-50 Sephadex RNA Columns (Roche) was employed to purify biotinylated RNA synthesized in vitro with the help of T7 RNA polymerase. Biotinylated miR-370-3p sense was named as bio-miR-370-3p-WT probe, and biotinylated miR-370-3p antisense was named as bio-miR-370-3p-MUT probe. Transfected cells were lysed with lysis buffer and incubated with streptavidin-coated magnetic beads to pull down the biotin-labeled RNA complex. Human Ago2 or normal mouse IgG antibody (Millipore) were used. The beads were washed and the precipitates were purified with TRIzol (Takara). The abundance of circAGFG1 was determined with qRT-PCR.

### Western blot analysis

Extracted proteins were separated by 10% SDS-PAGE, and then transferred onto PVDF membrane (Bio-Rad). After being blocked with 5% skimmed milk, the membranes were incubated with primary antibodies against RAF1, p-RAF1, MEK1/2, p-MEK1/2, ERK1/2, p-ERK1/2 and GAPDH at 4 °C. After overnight incubation and then incubated with secondary antibodies at room temperature for 2 h. All antibodies were purchased from Abcam (Burlingame, CA, USA). In the end, bands were measured with Immobilob™ Western Chemiluminescent HRP Substrate (Millipore).

### Statistical analysis

All independent experiments were conducted for three times. SPSS 19.0 software (IBM Corporation, Armonk, NY, USA) was responsible for data analyses. Data of three experimental results were exhibited as the mean ± standard deviation (SD). Student’s t-test and one-way ANOVA were two statistical methods for comparison of difference between two or more groups. P<0.05 is a symbol that indicates statistical significance.

## Results

### Downregulation of circAGFG1 restrained cell viability, proliferation and migration, and promoted cell apoptosis in cervical cancer

**Fig. 1:**
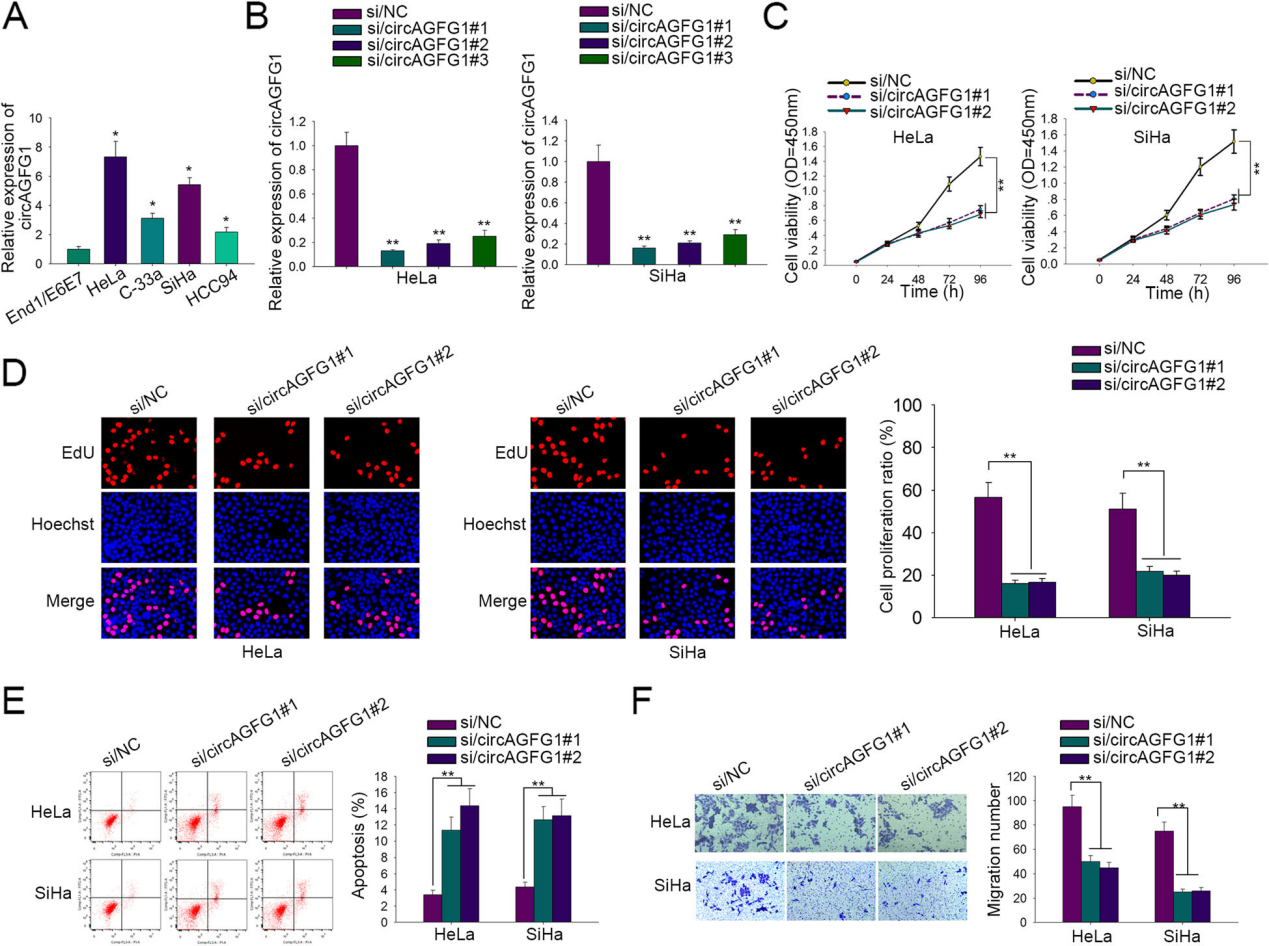
CircAGFG1 was overexpressed in cervical cancer and its downregulation restrained the proliferation and migration**. a** CircAGFG1 expression in cervical cancer cells (HeLa, C-33a, SiHa and HCC94) and normal cervical cell End1/E6E7 was measured by qRT-PCR. **b** Interference efficiency of three siRNAs containing si/circAGFG1#1/2/3 in HeLa and SiHa cells via qRT-PCR. CircAGFG1-silenced HeLa and SiHa cells were used for following mechanism assays. 0.1–1 pmol/150–300 ng of siRNA was used. **c**, **d** CCK-8 and EdU assays were performed in HeLa and SiHa cells to detect cell viability at 0, 24, 48, 72 and 96 h and cell proliferation. **e** Cell apoptosis was measured via flow cytometry analysis in HeLa and SiHa cells. **f** Transwell assay for cell migration ability in HeLa and SiHa cells. Data of three experimental results were exhibited as the mean ± standard deviation (SD). ^*^P < 0.05, ^**^P < 0.01

At first, the expression of circAGFG1 was separately tested in CC cell lines (HeLa, C-33a, SiHa and HCC94) and normal cervical cell line End1/E6E7. Results of qRT-PCR demonstrated that circAGFG1 was overexpressed in CC cells compared to normal cells. Moreover, HeLa and SiHa cells exhibiting the highest circAGFG1 expression were chosen for subsequent assays (Fig. 1a). Next, we explored the effect of circAGFG1 on biological function of CC cells. As illustrated in Fig. 1b, expression of circAGFG1 was apparently reduced after transfection of three specific siRNAs (si/circAGFG1#1/2/3) into cells, among which si/circAGFG1#1 and si/circAGFG1#2 with the best interference efficiency were selected for the function experiments. CCK-8 and EdU results showed that the viability and proliferation ability of CC cells was inhibited by circAGFG1 knockdown (Fig. 1c and d). As for cell apoptosis, flow cytometry analysis demonstrated that the apoptosis of CC cells was markedly boosted with circAGFG1 inhibition (Fig. 1e). In addition, transwell assay revealed that migration number of HeLa and Siha cells were decreased due to silencing of circAGFG1 (Fig. 1f). To summarize, circAGFG1 was upregulated in CC cells and its silencing inhibited the proliferation and migration in CC.

### MiR-370-3p was downregulated in CC and negatively modulated by circAGFG1

**Fig. 2:**
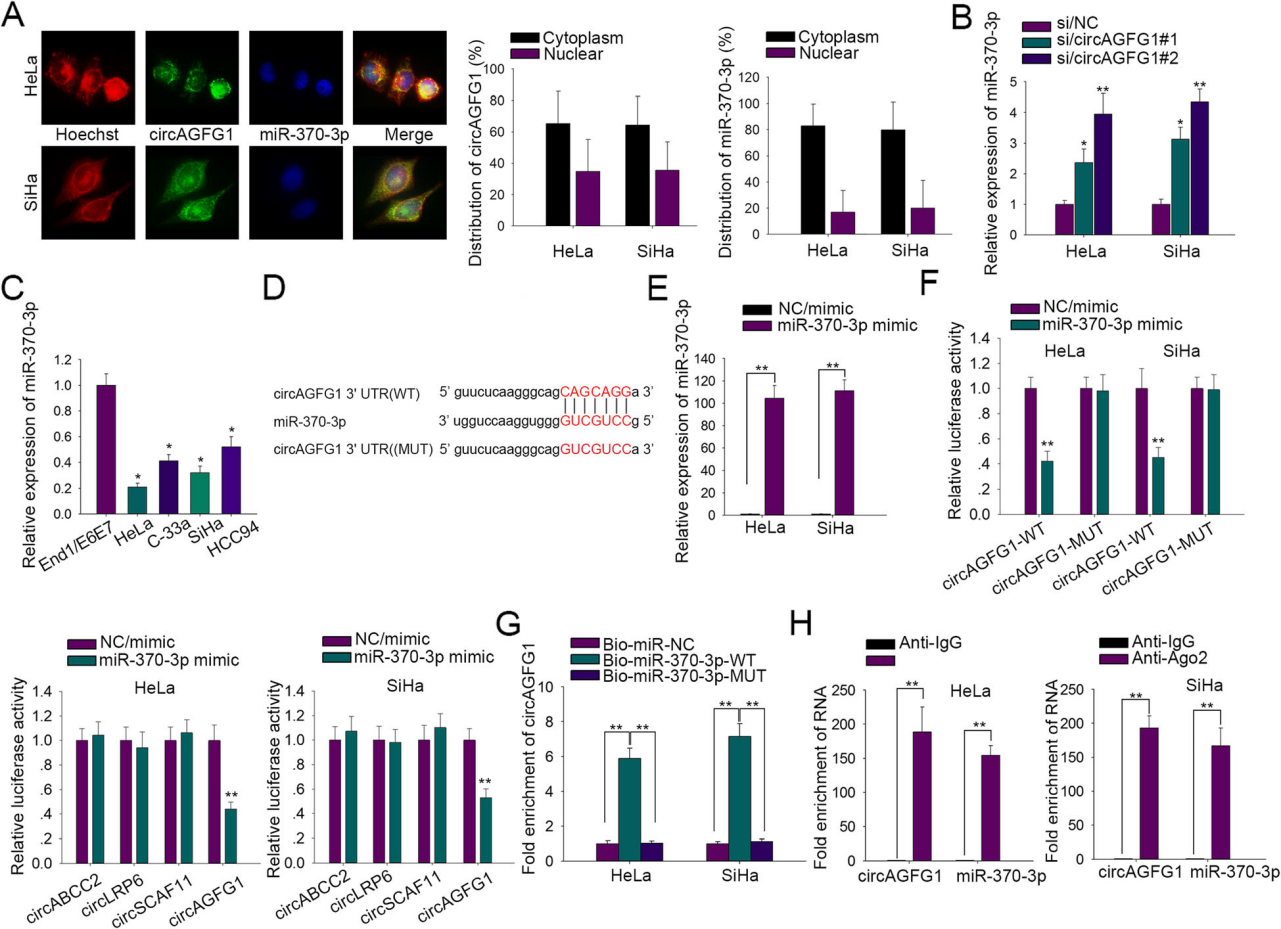
MiR-370-3p was downregulated in cervical cancer and negatively modulated by circAGFG1. **a** RNA-FISH images exhibiting the subcellular locations of circAGFG1 and miR-370-3p in HeLa and SiHa cells. The quantification results were shown on the right. **b** Influence of circAGFG1 knockdown using two siRNAs on miR-370-3p levels in HeLa and SiHa cells via qRT-PCR. **c** Low expression of miR-370-3p in cervical cancer cells was revealed via qRT-PCR, in comparison with End1/E6E7 cells. **d** The potential wild-type and mutated binding sites of circAGFG1 for miR-370-3p. **e**, **f** Overexpression efficiency of miR-370-3p mimic was determined by qRT-PCR, followed by luciferase reporter activity affirming the binding between circAGFG1 and miR-370-3p. The specific interaction between circAGFG1 and miR-370-3p compared with other circRNAs was also affirmed. These assays were implemented in HeLa and SiHa cells. (G-H) RNA pull down and RIP assays followed by qRT-PCR were conducted in HeLa and SiHa cells for the combination of circAGFG1 with miR-370-3p. Data of three experimental results were exhibited as the mean ± standard deviation (SD). ^*^P < 0.05, ^**^P < 0.01

To investigate the molecular mechanism of circAGFG1, we first discovered the potential miRNAs by using starBase v3.0 (http://starbase.sysu.edu.cn/). Among numerous candidates with strict stringency, miR-370-3p exhibited the highest increase in circAGFG1-silenced CC cells, compared with control cells (Additional file 1: Figure S1A). Previous studies have reported that miR-370-3p can function as a tumor suppresser and be absorbed by circRNAs in different kinds of cancers [11, 18, 19]. Here we carried out FISH assay to elucidate the subcellular location of circAGFG1 and miR-370-3p. Results presented that both circAGFG1 and miR-370-3p were localized in the cytoplasm (Fig. 2a). Knockdown of circAGFG1 could promote miR-370-3p expression, which meant circAGFG1 might negatively regulated miR-370-3p in CC (Fig. 2b). Furthermore, miR-370-3p was found to be downregulated in CC cells (Fig. 2c). To confirm the interaction between circAGFG1 and miR-370-3p, we first constructed circAGFG1-WT and circAGFG1-MUT luciferase reporter plasmids, and overexpressed miR-370-3p (Fig. 2d and e. Luciferase reporter assay revealed that overexpression of miR-370-3p only strikingly reduced the luciferase activity of wild type circAGFG1, except for that of mutant circAGFG1 (Fig. 2f). To validate the specificity of the interplaying between circAGFG1 and miR-370-3p, we firstly explored the effect of miR-370-3p overexpression on the luciferase activities of non-targets. It was affirmed that miR-370-3p up-regulation had no effect on the luciferase activities of non-targets (Fig. 2f). In addition, to exclude the possibility that circAGFG1 interacted with miR-370-5p, the star strand of miR-370-3p, we searched the sequence of miR-370-3p’s pre-miRNA from which the seed region sequence of miR-370-5p, miR-370-3p’s star strand, was attained, as shown in Additional file 1: Figure S1B. There were no reliable binding sites between miR-370-5p and circAGFG1 (MUT). These data validated the specificity of the binding of miR-370-3p to circAGFG1. Then RNA pull down assay demonstrated that circAGFG1 were merely pulled down by biotinylated miR-370-3p-WT but not by biotinylated miR-370-3p-MUT in CC cells, hinting the fold enrichment of circAGFG1 in Bio-miR-370-3p-WT group (Fig. 2g). Through RIP assay, we observed that circAGFG1 and miR-370-3p presented high enrichment in Ago2 group compared to IgG group, indicating the fold enrichment of circAGFG1 and miR-370-3p in a RNA-induced silencing complex (RISC) (Fig. 2h). Taken together, miR-370-3p was downregulated in CC and negatively regulated by circAGFG1.

### CircAGFG1 activated RAF/MEK/ERK pathway by sponging miR-370-3p

**Fig. 3:**
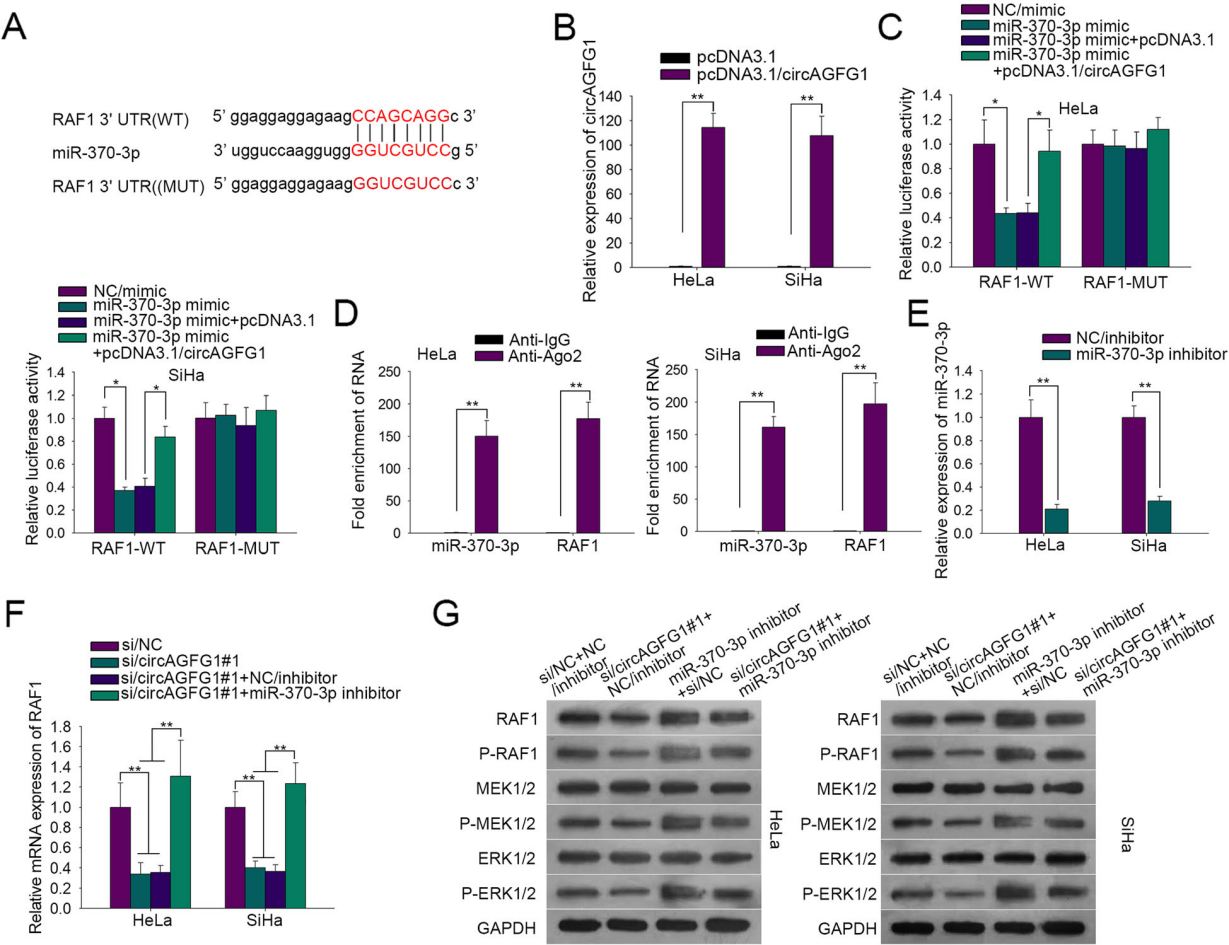
CircAGFG1 activated RAF/MEK/ERK pathway by sponging miR-370-3p. **a** The miR-370-3p binding site on RAF1 (WT) and mutant binding site. Additionally, the sequence of miR-370-5p was also displayed. **b** qRT-PCR detection of overexpression efficiency of pcDNA3.1/circAGFG1, which was carried out in HeLa and SiHa cells. **c** Luciferase reporter assay to elucidate the regulation of circAGFG1 and miR-370-3p on RAF1 in HeLa and SiHa cells. **d** In HeLa and SiHa cells, RIP and qRT-PCR experiments were utilized to validate the interplay between miR-370-3p and RAF1. **e** Interference efficiency of miR-370-3p inhibitor in HeLa and SiHa cells. **f** Impact of circAGFG1 and miR-370-3p on RAF1 expression at mRNA level, as estimated by qRT-PCR in HeLa and SiHa cells. **g** Impacts of circAGFG1 and miR-370-3p on the protein levels of RAF1 as well as RAF/MEK/ERK pathway markers containing p-RAF1, p-MEK1/2 and p-ERK1/2 in both HeLa and SiHa cells. Data of three experimental results were exhibited as the mean ± standard deviation (SD). ^*^P < 0.05, ^**^P < 0.01

After starBase v3.0 prediction, we acquired 37 shared mRNA targets of miR-370-3p (Additional file 1: Figure S1C). qRT-PCR examined the expression levels of these genes respectively in tumor cells and normal cells or in tumor cells with or without miR-370-3p up-regulation. Five genes were significantly high in tumor cells and 14 genes were significantly down-regulated in miR-370-3p-overexpressed tumor cells (Additional file 1: Figure S1D-E). Subsequently, Venn diagram was drawn and eventually gained the only one common gene: RAF1 (Additional file 1: Figure S1F). RAF/MEK/ERK signaling pathway has been confirmed to be promising therapeutic targets for cancer, among which RAF1 plays a key role in activating the phosphorylation of downstream MEK1/MEK2 and ERK1/ERK2 and promoting cancer progression [20]. Thereafter, we firstly sought to explore the feasible relationship between miR-370-3p and RAF1 in CC. The wild-type and mutated binding sites of RAF1, as well as the sequences of miR-370-5p were delineated in Fig. 3a. It was worth noting that no reliable binding affinity was found between miR-370-5p and RAF1 (MUT), excluding the possibility that the sequences of RAF1 (MUT) was not available (Additional file 1: Figure S1B). Overexpression of circAGFG1 by pcDNA3.1/circAGFG1 was detected via qRT-PCR in two cells (Fig. 3b). As demonstrated in Fig. 3c, miR-370-3p mimic apparently inhibited the luciferase activity of RAF1-WT, which was countervailed through adding pcDNA3.1/circAGFG1. However, the luciferase activity of RAF1-MUT was not effected by either miR-370-3p mimic or pcDNA3.1/circAGFG1, alone and in combination. These data proved the co-influences of miR-370-3p and circAGFG1 on RAF1 (Fig. 3c). Moreover, RIP assay showed the fold enrichment of miR-370-3p and RAF1 in Ago2 group compared to control group (Fig. 3d). Next, we explored the regulation of circAGFG1 and miR-370-3p on RAF1 expression. First, we inhibited miR-370-3p expression in HeLa and SiHa cells (Fig. 3e). Results of qRT-PCR showed that knockdown of circAGFG1 inhibited RAF1 mRNA expression, and the effect was abolished by miR-370-3p suppression (Fig. 3f). As shown in Fig. 3g, miR-370-3p suppression increased RAF1, p-RAF1, p-MEK1/2 and p-ERK1/2 protein expressions, and circAGFG1 knockdown lowered RAF1, p-RAF1, p-MEK1/2 and p-ERK1/2 protein expressions, which was restored by miR-370-3p inhibitor. There was no change in the whole protein of MEK1/2 and ERK1/2 (Fig. 3G and Additional file 1: Figure S1 G). In brief, circAGFG1 stimulated RAF/MEK/ERK pathway by sponging miR-370-3p.

### CircAGFG1 affected CC proliferation and migration via miR-370-3p/RAF1 axis

**Fig. 4:**
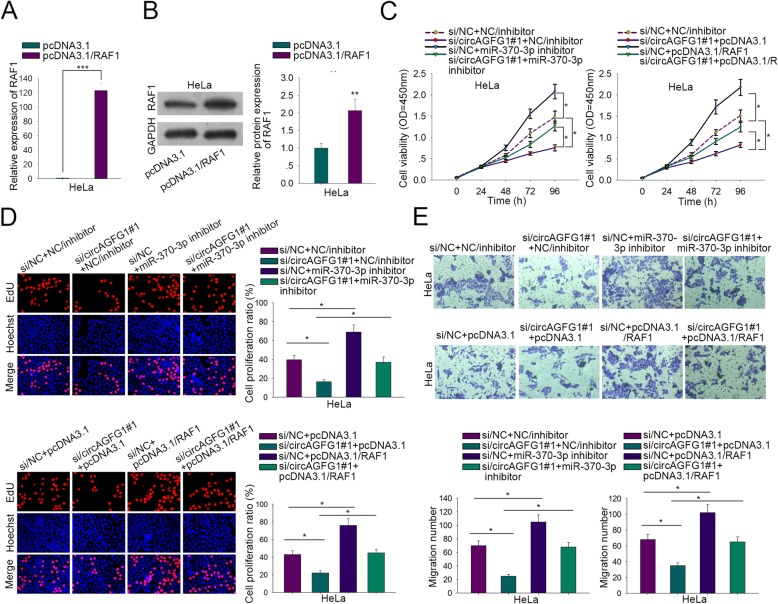
CircAGFG1 influenced CC proliferation and migration via miR-370-3p/RAF1 axis. **a**, **b** Transfection efficiency of pcDNA3.1/RAF1 in HeLa cells was verified via qRT-PCR and western blot. The right one in Fig. 2b was the quantification result. **c**, **d** CCK-8 and EdU analyses of the viability at 0, 24, 48, 72 and 96 h and proliferation properties of HeLa cells. **e** Transwell result of the migration property in HeLa cells. Data of three experimental results were exhibited as the mean ± standard deviation (SD). ^*^P < 0.05, ^***^P < 0.001

To investigate whether circAGFG1 influenced the biological functions of CC cells through miR-370-3p/RAF1 axis, we performed rescue assays in HeLa cells. RAF1 was overexpressed by pcDNA3.1/RAF1 in both mRNA and protein levels (Fig. 4a and b). Silencing of circAGFG1 markedly repressed cell viability but the effect was reversed by inhibition of miR-370-3p or overexpression of RAF1. In addition, both miR-370-3p inhibition and RAF1 overexpression could accelerate cell viability (Fig. 4c). The same results of EdU assay for cell proliferation were shown in Fig. 4d. In flow cytometry analysis, circAGFG1 silencing-induced cell apoptosis was weakened by repressed miR-370-3p or raised RAF1. Also, miR-370-3p depletion or RAF1 addition could decrease cell apoptosis (Additional file 1: Figure S1H). The migratory ability was suppressed when circAGFG1 was silenced, but miR-370-3p inhibition or RAF1 upregulation recovered the phenomenon. Moreover, cell migration was facilitated through miR-370-3p inhibition or RAF1 upregulation (Fig. 4e). Collectively, circAGFG1 influenced CC proliferation and migration via miR-370-3p/RAF1 axis.

## Discussion

Competitive endogenous RNA (ceRNA) ceRNAs can act like a molecular sponge of miRNAs, hence reducing miRNAs-regulated inhibition of the target genes [21]. In 2013, circRNAs were reported to be a key subtype of ceRNAs that functioned to absorb miRNAs and abrogated its impact on downstream gene expression [22, 23]. It is evident that circRNAs affect not only occurrence and development of cervical cancers, but also those of other cancers. In lung cancer, circular RNA hsa_circRNA_103809 acts as an oncogene through miR-4302/ZNF121/MYC axis [24]. CircRNA_102171 drives papillary thyroid cancer cell proliferation, migration and invasion via activating CTNNBIP1-dependent β-catenin pathway [25]. CircRNA_100782 promotes pancreatic cancer proliferation through the IL6-STAT3 pathway via sponging miR-124 [26]. In breast cancer, hsa_circRNA_0006528 activates the MAPK/ERK signaling pathway by absorbing miR-7-5p [27].

Non-coding RNAs are RNAs that have no capability of encoding proteins. Except for long non-coding RNAs (lncRNAs), circRNAs could also compete with protein-coding mRNAs for binding to microRNAs (miRNAs) in ceRNA networks [28]. Herein, we found circAGFG1 (ID: hsa_circ_0058514) that develops carcinogenic impacts on cellular function of triple-negative breast cancer and non-small cell lung cancer [9, 10]. Our study firstly disclosed that circAGFG1 was heightened in CC and circAGFG1 silencing impaired the proliferation and migration properties of CC.

Among assumed targets of circAGFG1, we chose miR-370-3p for further exploration. The well-known tumor inhibitor role of miR-370-3p has been revealed in numerous tumors. For instance, CXCL12-regulated miR-370–3p acts as a tumor suppressor of nonfunctional pituitary adenomas via targeting HMGA2 [29]. LncRNA H19 enhances TGF-β-activated epithelial-mesenchymal transition (EMT) in ovarian cancer through serving as a ceRNA of miR-370-3p [30]. Overexpression of miR-370-3p impairs glioblastoma multiforme resistance to temozolomide via affecting MGMT expression [31]. Then it was observed that circAGFG1 and miR-370-3p were situated in the cytoplasm, and miR-370-3p was negatively modulated by circAGFG1. Further, through qRT-PCR screening, we found the target gene: Raf-1 proto-oncogene, serine/threonine kinase (RAF1), a member in RAF/MEK/ERK pathway. RAF/MEK/ERK pathway is a noted oncogenic pathway functioned in a big amount of cancers [32–34]. Besides, RAF1 has been indicated to exert carcinogenic property in various carcinomas [14–16]. Consistently, mechanism experiments affirmed that circAGFG1 exhausted miR-370-3p to regulate RAF1 expression and further activate RAF/MEK/ERK pathway. The relationship among circAGFG1, miR-370-3p and RAF1 was firstly displayed by our research.

In the end, rescue assays certified that circAGFG1 promoted CC proliferation and migration via miR-370-3p/RAF1 axis. These findings provided a potential biomarker and an admissible therapeutic target for CC patients. Nevertheless, other modulation mechanisms of circAGFG1 in cervical cancer remain to be discovered.

## Conclusion

In summary, this study revealed that circAGFG1 exerted oncogenic properties in cervical cancer by sponging miR-370-3p to upregulate RAF1. Our research findings potentially help to provide an admissible therapeutic target for CC patients.

## Supplementary information

**Additional file 1. Figure S1.** (A) The heatmap plotted through the results from qRT-PCR told us that miR-370-3p was the most up-regulated miRNA in response to circAGFG1 down-regulation in CC cells. (B) The sequences of miR-370-3p’s pre-miRNA and miR-370-3p’s star strand miR-370-5p not sufficiently matched for circAGFG1 (MUT) or RAF1 (MUT). (C) 37 mRNA targets were gained from the intersection of microT, miRmap and PITA. (D-F) The Venn diagram of two pie chart screened out the downstream factor: RAF1. (G) The quantized data of the protein levels of RAF1 and RAF1/MERK/ERK pathway downstream genes in both HeLa and SiHa cells. (H) Rescue assays on cell apoptosis were carried out through flow cytometry analyses in HeLa cells. Data of three experimental results were exhibited as the mean ± standard deviation (SD). ^*^P < 0.05, ^**^P < 0.01.

## Data Availability

Research Data are not shared.

## Abbreviations

ATCC: American Type Culture Collection
CC: Cervical cancer
ceRNA: competitive endogenous RNA
circRNAs: circular RNAs
DMEM: Dulbecco’s Modified Eagle’s Medium
EdU: 5-Ethynyl-2′-Deoxyuridine
EMT: Epithelial-mesenchymal transition
FBS: Fetal bovine serum
lncRNAs: Long non-coding RNAs
miRNAs: microRNAs
PI: Propidium iodide
RAF1: Raf-1 proto-oncogene, serine/threonine kinase
RIP: RNA immunoprecipitation
