# Supplementary figures and images for: CircAGFG1 promotes cervical cancer progression via miR-370-3p/RAF1 signaling
Source: BMC Cancer. 2019 Nov 8;19:1067. doi: 10.1186/s12885-019-6269-x (PMC6842182; doi:10.1186/s12885-019-6269-x)

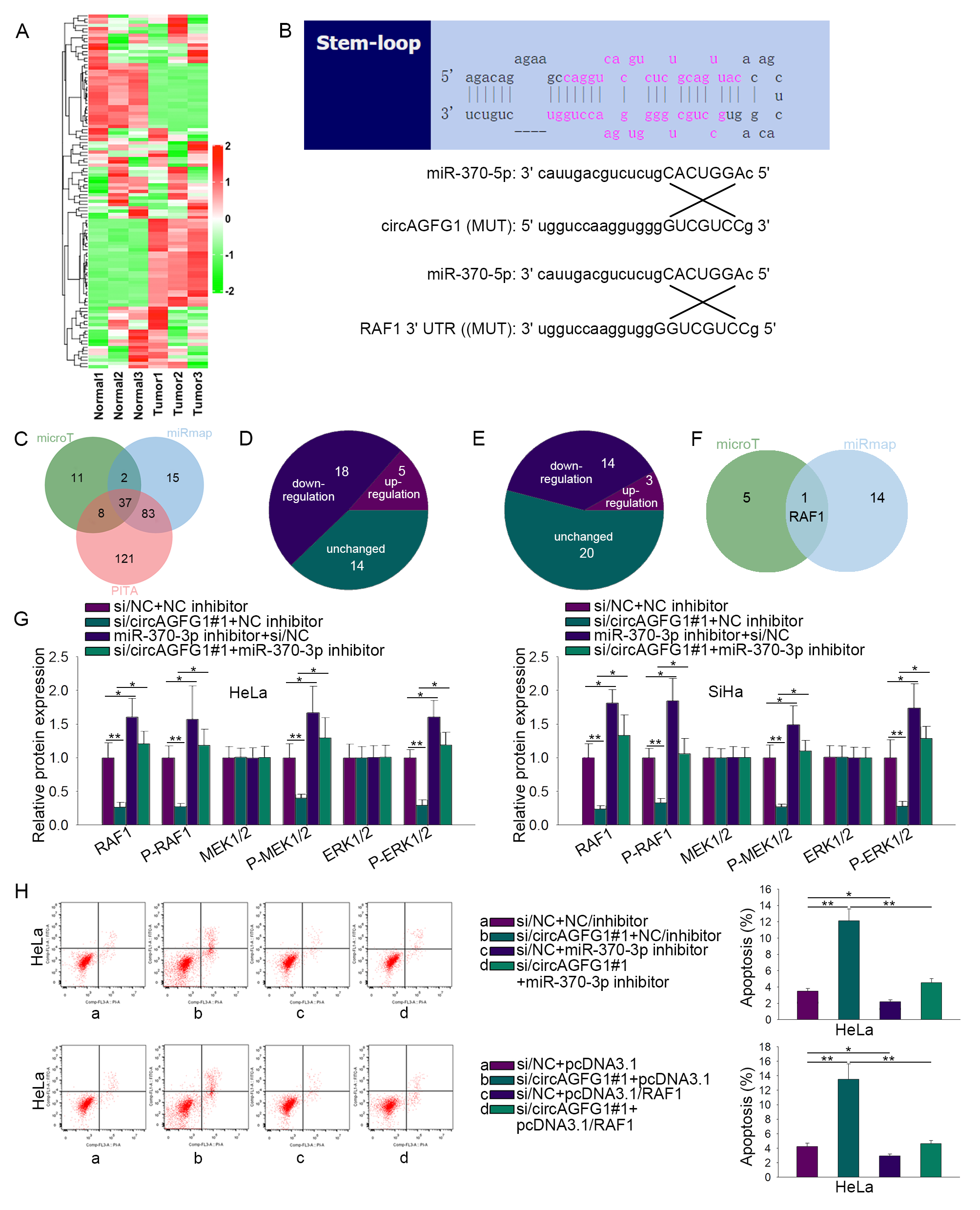

Supplement: Supplementary file 1 — Additional file 1. Figure S1. (A) The heatmap plotted through the results from qRT-PCR told us that miR-370-3p was the most up-regulated miRNA in response to circAGFG1 down-regulation in CC cells. (B) The sequences of miR-370-3p’s pre-miRNA and miR-370-3p’s star strand miR-370-5p not sufficiently matched for circAGFG1 (MUT) or RAF1 (MUT). (C) 37 mRNA targets were gained from the intersection of microT, miRmap and PITA. (D-F) The Venn diagram of two pie chart screened out the downstream factor: RAF1. (G) The quantized data of the protein levels of RAF1 and RAF1/MERK/ERK pathway downstream genes in both HeLa and SiHa cells. (H) Rescue assays on cell apoptosis were carried out through flow cytometry analyses in HeLa cells. Data of three experimental results were exhibited as the mean ± standard deviation (SD). *P < 0.05, **P < 0.01. [file 12885_2019_6269_MOESM1_ESM.tif]
